# The target trial framework in global health research: barriers and opportunities

**DOI:** 10.7189/jogh.15.03014

**Published:** 2025-03-21

**Authors:** Ali Al-kassab-Córdova, Esteban A Alarcón-Braga, Camila Olarte Parra, Niveditha Devasenapathy, Martin Gerdin Wärnberg, Anthony A Matthews

**Affiliations:** 1 Unit of Epidemiology, Institute of Environmental Medicine, Karolinska Institutet, Solna, Stockholm, Sweden; 2 Centro de Excelencia en Estudios Económicos y Sociales en Salud, Universidad San Ignacio de Loyola, Lima, Peru; 3 Facultad de Ciencias de la Salud, Universidad Peruana de Ciencias Aplicadas, Lima, Peru; 4 George Institute for Global Health, India; 5 Department of Global Public Health, Karolinska Institutet, Solna, Sweden; 6 Perioperative Medicine and Intensive Care, Karolinska University Hospital, Solna, Sweden

## Abstract

A randomised trial is the best way to make causal inferences when evaluating the effectiveness and safety of health interventions in global health research. Trials, however, are inherently expensive, unfeasible in many scenarios, and may raise ethical issues. In these scenarios, we must turn to analyses of observational data to learn what works. The target trial framework provides an organising principle for the design of observational studies that can lead to clinically interpretable results and analytic approaches that can reduce common biases. In this analysis, we describe the global distribution of data sources used in applications of the target trial framework and discuss barriers to its increased use in global health research, such as limited access to high-quality observational data. We then suggest a cost-effective solution of incorporating the collection of additional high-quality observational data into the implementation of large randomised trials in low- and middle-income countries. We found that the target trial framework is underutilised in observational studies conducted in most low- and middle-income countries. The main barriers are little available data and few trained researchers, which can be overcome by incorporating high-quality observational data collection into the data collection phase of large randomised trials, and by introducing small adjustments to the teaching curriculum.

Establishing cause-and-effect relationships is pivotal in global health; it highlights opportunities for interventions by identifying the need and potential to implement actions that either minimise exposure to harmful factors or enhance exposure to beneficial ones. Yet, drawing causal inferences is complex.

A randomised trial is the best way to make causal inference when evaluating the effectiveness and safety of a health intervention. Trials, however, are inherently expensive, unfeasible in many scenarios, and may raise ethical issues [1]. There are additional barriers to conducting a trial in low- and middle-income countries (LMICs) compared with high-income settings, including less financial support and human capacity, infrastructure that makes practical implementation challenging, and occasionally inadequate regulatory oversight [2,3]. For example, many countries lack a central ethics body to approve trial protocols, requiring independent approval at each recruiting hospital. This process varies between private and governmental hospitals, further hindering the trial and underscoring the fragmentation of healthcare systems [4]. Besides, as funding for clinical trials in LMICs comes predominantly from high-income countries [5], locally relevant research questions may be understudied, despite the need for better interventions. Analyses of observational data can, therefore, play a key role in guiding decision making in LMIC, although such data are difficult to come by in many settings. Hence, identifying cost-effective and scalable solutions approaches is paramount to guide decision-making in resource-limited settings.

Using observational data to make causal inferences, however, is challenging. Not only is there the inherent problem of confounding, but suboptimal study design decisions can also result in severe bias [6]. The target trial framework provides an organising principle for the design of observational studies that can lead to clinically interpretable results and analytic approaches that can reduce common biases [1,7,8]. The framework comprehends a two-step process: first, the articulation of the causal question through the specification of the protocol of a hypothetical randomised trial, *i.e.* the target trial; second, the emulation of the target trial using observational data and appropriate methods. Explicit target trial emulation means the only unavoidable difference between the target trial and its emulation is how treatment is assigned to eligible individuals at the start of follow-up: at random in the target trial and under routine practice in the emulation.

Unlike traditional observational analyses, the target trial emulation approach is designed to answer causal questions by framing observational analyses within the paradigm of a randomised clinical trial. Consequently, it follows the parameters of a hypothetical clinical trial and addresses bias through a transparent structured study design approach. The rigorous use of observational data enables effect estimation in broader populations that are often under or not represented in clinical trials, such as marginalised social and ethnic groups, the elderly, and patients with rare conditions, among others. Similarly, long-term outcomes – often challenging to evaluate in traditional trials – can also be assessed, allowing for the observation of rare or delayed outcomes that might otherwise be missed.

Four recent reviews have shown an upward trend of published observational studies that use the target trial framework to guide study design, but none reported the countries in which the studies were conducted [9–12]. Understanding the global distribution of studies is essential to identify disparities and guide efforts to strengthen research capacity in developing countries. We therefore aimed to carry out a scoping review to understand the global distribution of authors and data sources used in studies applying the target trial framework. We will then discuss the barriers to increased use of the target trial framework in global health research, why it provides a unique opportunity to generate well-designed, affordable, observational evidence, and finally provide suggestions of how uptake of this approach can be achieved globally.

## SCOPING REVIEW

We conducted a scoping review of the literature in accordance to the PRISMA-ScR checklist [13,14]. We reused the strategy from the most recent scoping review on this topic, which incorporated a controlled vocabulary (*e.g.* MeSH, Emtree) alongside relevant keywords to represent the concept of ‘target trial emulation’ [9] (Table S1 in the **Online Supplementary Document**). We then applied this strategy to MEDLINE (via Ovid), PROSPERO, Embase, Cochrane Library, and Scopus, from inception to 11 June 2024.

We included peer-reviewed observational studies explicitly reporting the utilisation of the target trial framework, without restrictions on population, interventions, outcomes, or language. We excluded conference abstracts, preprints, articles that used simulated data, editorials, letters to the editor, and reviews. We imported the resulting database entries into the Rayyan review management software [15], where we eliminated duplicates and performed the screening. Specifically, two researchers (AAC and EAB) independently screened the retrieved studies, resolving discrepancies by consensus. In terms of agreement, we obtained a Cohen’s kappa of 0.7076.

Both reviewers independently extracted the following information from included studies: authors, title, country, year of publication, and country of affiliation of first and senior authors. Income classifications for the countries studied were determined using the World Bank’s income categorisation system [16].

## FINDINGS

**Figure 1:**
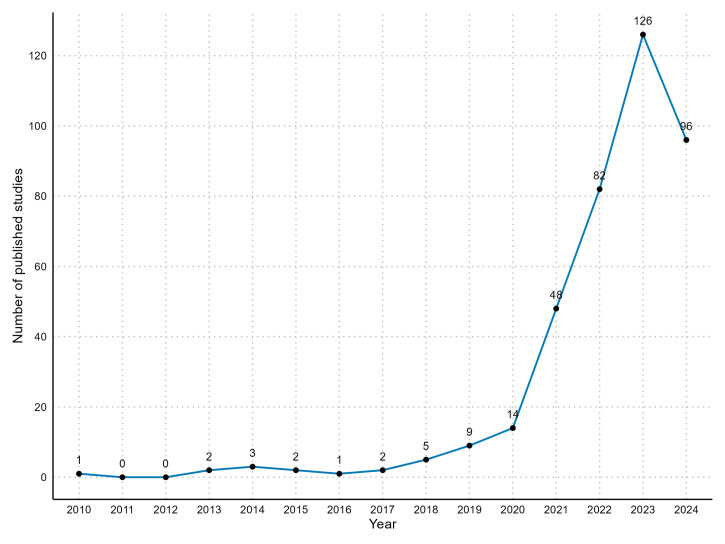
Trend of target trial emulation publications from 2010 to 2024.

We identified 1432 non-duplicate records for title/abstract evaluation, of which 480 passed to full-text assessment. Subsequently, 391 met the selection criteria and were included in the full review (Figure S1 in the **Online Supplementary Document**). The annual utilisation of the target trial framework for observational analyses has increased from 14 published studies in 2020 to 126 in 2023 (**Figure 1**). Most of this increase was driven by studies using data from high-income countries (Figure S2 in the **Online Supplementary Document**).

### Geographic trends

**Figure 2:**
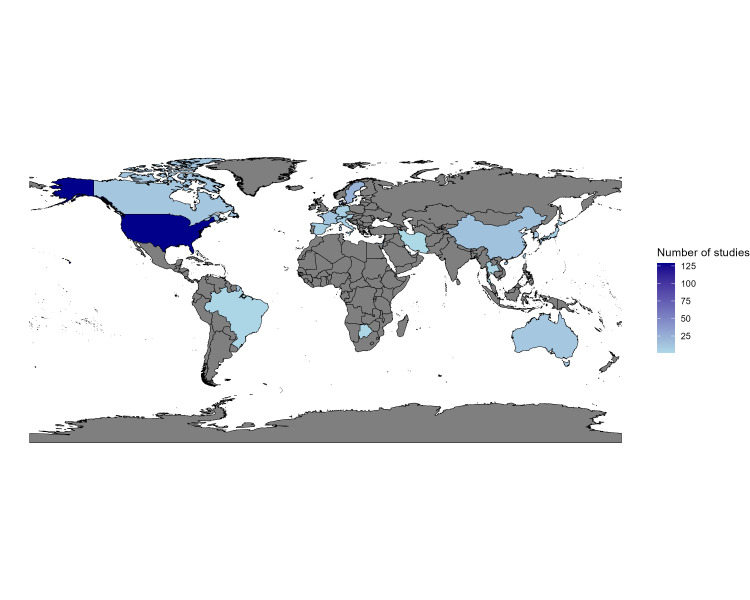
Global distribution of the target trial framework publications.

The data used in these studies were from Australia (n = 10), Austria (n = 3), Botswana (n = 2), Brazil (n = 2), Canada (n = 11), China (n = 14), Denmark (n = 23), France (n = 13), Germany (n = 7), Iran (n = 1), Ireland (n = 1), Israel (n = 6), Italy (n = 6), Japan (n = 5), Qatar (n = 1), Singapore (n = 1), South Korea (n = 10), Spain (n = 6), Sweden (n = 24), Switzerland (n = 5), Taiwan (n = 9), Thailand (n = 3), The Netherlands (n = 5), UK (n = 42), USA (n = 147), while the rest (n = 34) were multi-country studies (**Figure 2**). Studies using data from the USA, the UK, Denmark, and Sweden (all high-income countries) accounted for over half (60%) of all studies that utilized the target trial framework worldwide. Botswana, Brazil, China, Iran, and Thailand are the only LMICs from which data have been used in applications of the target trial framework (6%). Twelve multi-country studies also included data from LMICs.

### Author demographics

Twenty-five studies had a first author (6%) and 16 studies had a senior author (4%) from a LMIC. In the multi-country studies, all first and last authors were affiliated with institutions based in high-income countries.

### Limitations

Some limitations should be acknowledged in interpreting our findings. We did not include regional databases, which could have led to an underrepresentation of studies published in regional journals not indexed in the major databases we searched. This likely contributed to an overrepresentation of articles using data from high-income countries. While several studies explicitly reported the utilisation of the target trial framework, this does not guarantee that the framework was correctly implemented. Finally, the exclusion of preprints, conference abstracts, and non-peer-reviewed literature may have resulted in a failure to capture recent studies.

## WHY HAVE SO FEW APPLICATIONS OF THE TARGET TRIAL FRAMEWORK USED DATA FROM LOW- AND MIDDLE-INCOME COUNTRIES?

Although there has been a sharp increase in the use of the target trial framework to guide observational study design over the last decade, the global distribution of data used in these studies is highly skewed towards high-income countries. This could wholly be a consequence of fewer observational studies that aim to make causal inferences arising from LMICs. Regardless, the scoping review has highlighted two main challenges to address when attempting to increase the use of the target trial framework in these countries: the scarcity of reliable data sources and the lower number of researchers with the required expertise.

Most applications of the target trial framework use routinely collected data from electronic health records, health care registers, or insurance claims. It has been shown that these data sources can reliably capture the information required to make valid causal inferences for many important clinical questions, *i.e.* by having detailed information on baseline confounders and enabling prospective collection of outcomes [17]. It is, however, known that such data are often lacking or of poor quality in LMICs [2]. Challenges to collect high-quality data stem from planning (competent project managers, identification of stakeholders, setting objectives and a technical plan, and developing a timeline and estimated budget), the human element (information technology resistance, availability of skilled personnel, expertise, quality issues, and team make-up), well-defined processes (registry governance, data and access policy, and dissemination framework), and technical design (hardware and infrastructure, overall architecture, registry design, security, tools for reporting, and quality assurance) [3,18–20]. Another challenge is the fragmentation of health systems, in which patients often consult multiple health care providers at their discretion. As a result, implementing electronic health records that effectively track patient data becomes difficult [21]. Disparities in quality of data across LMICs can affect the ability to address locally relevant problems and reduce the generalisability of findings. This emphasises the need for a urgent and concerted effort to improve collection and archiving of electronic health records [22]. Without easy and affordable access to high-quality data, it is impossible for researchers to carry out well-designed, bias-free observational studies.

Implementation of the target trial framework, of course, requires trained researchers with the relevant methodological expertise, both epidemiological and statistical. In recent years, classroom teaching of causal inference has been centred around graduate programmes in Europe and the USA. The flow of knowledge to LMICs, therefore, requires a large financial investment to send researchers to study at these programmes and incentives to attract them back to their home countries to share and implement knowledge. Although there have been clear efforts to increase the methodological capacity of researchers from LMICs [23], it is hard to compete with the financial might and name-recognition of many institutions in high-income countries to retain the most skilled researchers. It is also difficult to fully appreciate, based on the available data, to what extent the disparities in authorship revealed here stem from discrepancies in the availability of the required expertise, as opposed to poor recognition of contributors from LMICs. However, the authorship patterns in the target trial literature closely resembles the patterns in other fields [24,25] and highlights the need for efforts to promote and ensure equitable collaborations and authorships [26].

## WHAT BENEFITS CAN INCREASED USE OF THE TARGET TRIAL FRAMEWORK BRING TO GLOBAL HEALTH RESEARCH?

The main advantages of the target trial framework for global health research are identical to the advantages of its use in other settings. First, if a causal question cannot be translated into the design of a hypothetical randomised trial, the question itself is not well defined. Designing a target trial before emulating it in observational data, therefore, facilitates articulating a precise causal question, which leads clinically interpretable evidence to support a decision problem. Second, in randomised trials, by design, the time an individual becomes eligible coincides with assignment to a treatment group and start of follow-up (time zero). However, it is easy to misspecify time zero when designing an observational study, and, for example, start following individuals and counting outcomes many years after they initiated treatment. Such misspecifications can lead to erroneous conclusions due to selection of artificially healthy individuals that survive and are event free from the time of initiation to the arbitrary time when follow up is started [27]. Third, even when a randomised trial is conducted, it cannot answer all clinically important questions. Observational analyses that use the target trial framework can, therefore, fill evidence gaps left by trials such as treatment effects over long follow up and within subpopulations excluded or underrepresented in trials.

There are, however, additional advantages to global health research that perhaps outweigh those found in other settings. Randomised trials conducted in LMICs must address barriers to obtaining informed consent that are less prevalent in other settings, such as illiteracy, cultural hesitation, language obstacles, and misunderstandings about trial procedures [28,29]. These trials are, therefore, usually carried out in very selected populations that are willing and able to give informed consent [30]. A consequence of this selection is evidence that is perhaps unrepresentative, meaning the treatment effect estimated within those that were eligible and gave informed consent may not reflect the true treatment effect in the ‘target population’ of everyone eligible to participate [31]. Observational analyses are more likely to be representative of the target population as data usually arise from sources such as electronic health records and issues of informed consent are dealt with at the data collection stage. The question then becomes, do the available data contain all the information needed to address confounding?

## HOW CAN WE INCREASE THE UTILISATION OF THE TARGET TRIAL FRAMEWORK IN GLOBAL HEALTH RESEARCH?

The target trial framework has the potential to improve the quality of observational studies that aim to support important treatment and policy decisions in LMICs. Increased capacity, however, is not possible without addressing the challenges regarding available data and trained researchers outlined above.

### Optimising the data collection process allows answering many research questions

As mentioned previously, the are several barriers that hinder the data collection process and are hard to overcome, mainly in developing countries [3,18–20]. Addressing these concerns requires intersectoral and multisectoral collaboration not only in the public sector, but also with private stakeholders. At the organisational level, this includes continuous in-service training, supportive supervision in the use of electronic databases, and mechanisms to review data quality. Additionally, the adaptability of digital records and the quality of electronic health record data can be enhanced by employing dedicated personnel to manage digitisation, thereby alleviating the burden on already stretched health care professionals. On an individual level, fostering behaviours that improve perceived confidence and competence among health care professionals is essential for successful implementation. Moreover, data-sharing policies and clear guidance for obtaining consent to use routine clinical data in research should be established to ensure ethical and effective data utilisation.

On top of that, secondary data analysis is not always a straightforward process and often requires foresight by those collecting the data. Large collaborative research initiatives such as GlobalSurg and the African Perioperative Research Group (APORG) have been launched to combine individuals’ data from all around the world – wherever they meet the study selection criteria – to improve the representativeness of the estimates, enhance the robustness of the findings, and facilitate a more comprehensive understanding of global health trends [32,33]. Integrating data from multiple sources into one dataset requires local, national, and international collaboration between researchers and data managers.

### A pragmatic approach to collecting observational data: integrate the process with the conduct of randomised trials

The best advice is to collect more and better health care data, *i.e.* through setting up comprehensive health care registers. However, the initial costs of such endeavours are monumental, and it will take many years until the benefits of the investment are realised. These funds will, therefore, likely be directed to randomised trials that provide answers to important questions that require immediate answers. Funding of these trials, however, provides a unique opportunity to collect observational data. Although this would impose an additional cost for randomised trials, by collecting such data, we could respond different causal questions beyond the index trial.

Take the recent E-MOTIVE cluster randomised trial, in which data from 78 hospitals in Kenya, Nigeria, South Africa, and Tanzania were used to estimate the effect of a bundle intervention during vaginal birth delivery that aimed to detect and prevent of postpartum haemorrhage (blood collection drape, uterine massage, oxytocic drugs, tranexamic acid, intravenous fluids, examination, and escalation). In the trial, 210 132 individuals underwent vaginal delivery and were randomised at the hospital level. Data were collected on the intervention, hospital characteristics, patient characteristics (*e.g.* delivery with forceps or vacuum), and the availability of essential drugs for postpartum haemorrhage (*e.g.* oxytocin and tranexamic acid). Outcome data were also collected on severe postpartum haemorrhage, postpartum laparotomy for bleeding, and maternal death from bleeding (as well as several other secondary outcomes) [34].

Large trials like E-MOTIVE require significant investment to set up a data collection infrastructure. Use of this infrastructure to collect additional data at baseline and over follow up would open up the opportunity to answer a plethora of other questions. For example, it could help understand the effect of delivery with forceps or vacuum on the risk of severe perineal and cervical lacerations and its consequences such as pelvic pain, sexual problems, and anal incontinence. There is evidence that the use of forceps or vacuum during delivery can increase the risk of maternal trauma, but most evidence is from high-income countries [35]. It would be possible to design and emulate a target trial to ask questions on the effect of forceps or vacuum using data collected from the E-MOTIVE trial if additional baseline data were collected on the demographic and clinical characteristics to adjust for confounding, and relevant outcomes over longer follow up. This would provide vital evidence in a large population that is under-researched at a fraction of the cost of a new randomized trial.

As an example, a recent observational study, nested within the ALERT trial, was conducted leveraging its infrastructure and data collection [36]. The ALERT was a cluster-randomised stepped-wedge trial conducted across 16 hospitals in Benin, Malawi, Tanzania and Uganda, aimed at improving intrapartum care through evidence-based practices to reduce perinatal mortality and morbidity [37]. By combining clinical data collected in this trial with ambient temperature data, a subseqeuent observational study sought to evaluate whether extreme heat is associated to early perinatal deaths [36]. Leveraging clinical trial infrastructure can provide a powerful framework to address numerous causal questions.

### High-quality causal inference teaching in low- and middle-income countries

The above strategy to increase the collection of high-quality observational data is worthless unless there are trained researchers that understand how the target trial framework can be used to design bias-free observational studies. Luckily, the target trial framework democratises causal inference as the knowledge required to understand and apply it is already part of the epidemiology curriculum at many universities.

A hurdle to understanding many novel causal inference methods, like the g-methods, has been the need for prior advanced training in statistics and mathematics [38]. However, the target trial framework requires nothing more than a basic understanding of how to design randomised trials, which is already included in the related teaching. The target trial framework is, therefore, a logical transition from teaching clinical trials to observational studies [39].

Incorporation of the target trial framework into the curriculum of medical research education in LMICs is, therefore, a low-hanging fruit that can quickly improve knowledge without considerable investment in fully designing curriculums. Additionally, stakeholders – including local research institutions, universities, health ministries, and international development organisations – should work together to develop training programmes for researchers, such as freestanding courses. Gaining buy-in from evidence-based practitioners on the target trial framework utilisation will facilitate its adoption and encourage contributions to further research.

## CONCLUSIONS

The target trial framework is underutilised in observational research conducted in most LMICs. The main barriers – the scarcity of data and trained researchers – must be addressed as immediate priorities to facilitate the effective adoption of this framework. These challenges can be overcome by incorporating high-quality observational data collection into the data collection phase of large randomised trials, and by implementing small adjustments to the teaching curriculum which would help build long-term capacity, equipping future researchers with the skills to ensure sustainable research. Local and international stakeholders should also collaborate to organise training opportunities for researchers. Further, fostering international collaborations for technical support, mentorship, and shared data repositories will be paramount in strengthening research capacity and promoting the widespread adoption of this framework. With these changes, increased utility of the target trial framework can improve the quality of observational studies that aim to support important treatment and policy decisions in developing countries.

## Additional material


Online Supplementary Document
