# Supplementary material for: The target trial framework in global health research: barriers and opportunities
Source: J Glob Health. 2025 Mar 21;15:03014. doi: 10.7189/jogh.15.03014 (PMC11926579; doi:10.7189/jogh.15.03014)
Supplement: Online Supplementary Document [file jogh-15-03014-s001.pdf]

Supplement to: l-kassab-Córdova A, Alarcón-Braga EA, Olarte Parra C, Devasenapathy N, Wärnberg MG, Matthews AA. The target trial framework in global health research: barriers and opportunities. J Glob Health. 2025;15:03014.

Figure S1. PRISMA flow diagram.

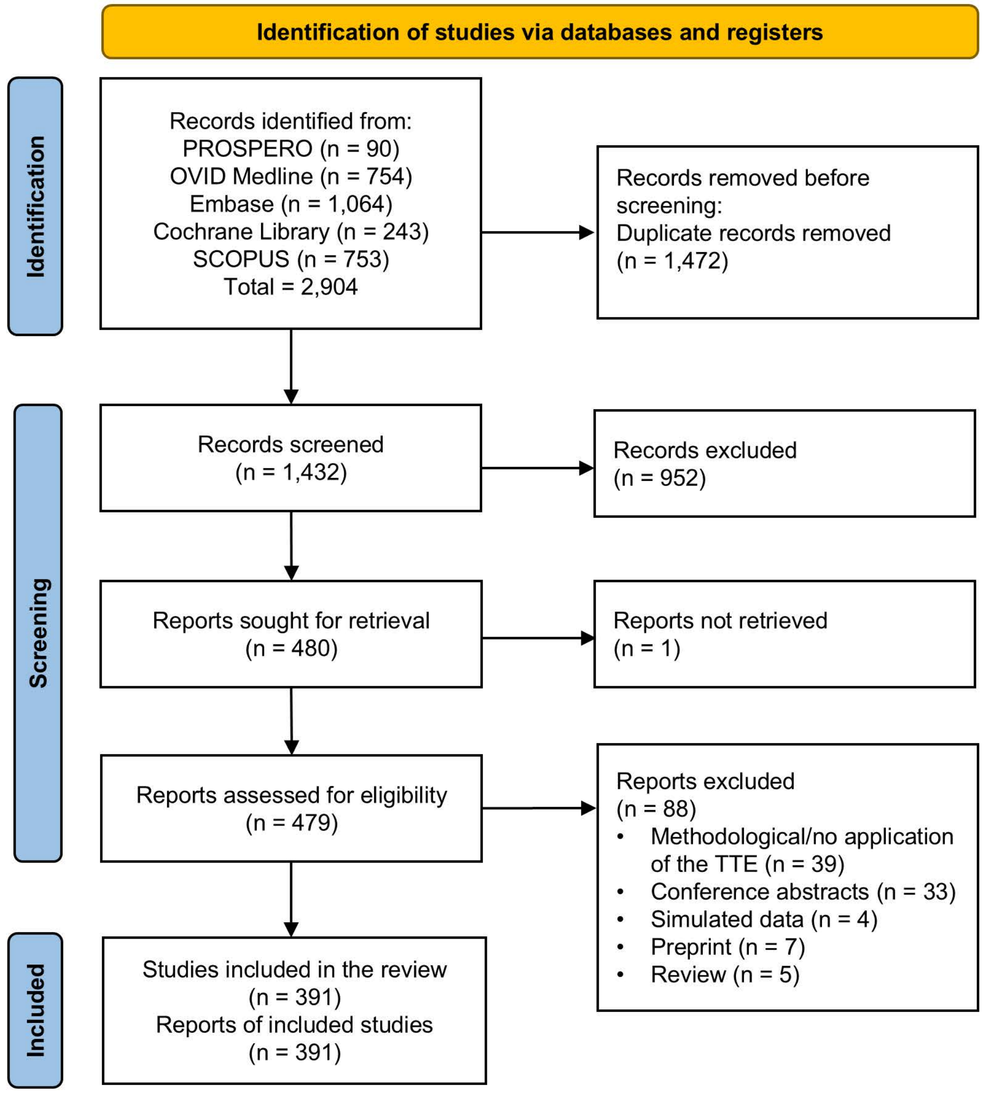

Figure S2. Trends of target trial emulation publications from 2010 to 2024, stratified by country income level.

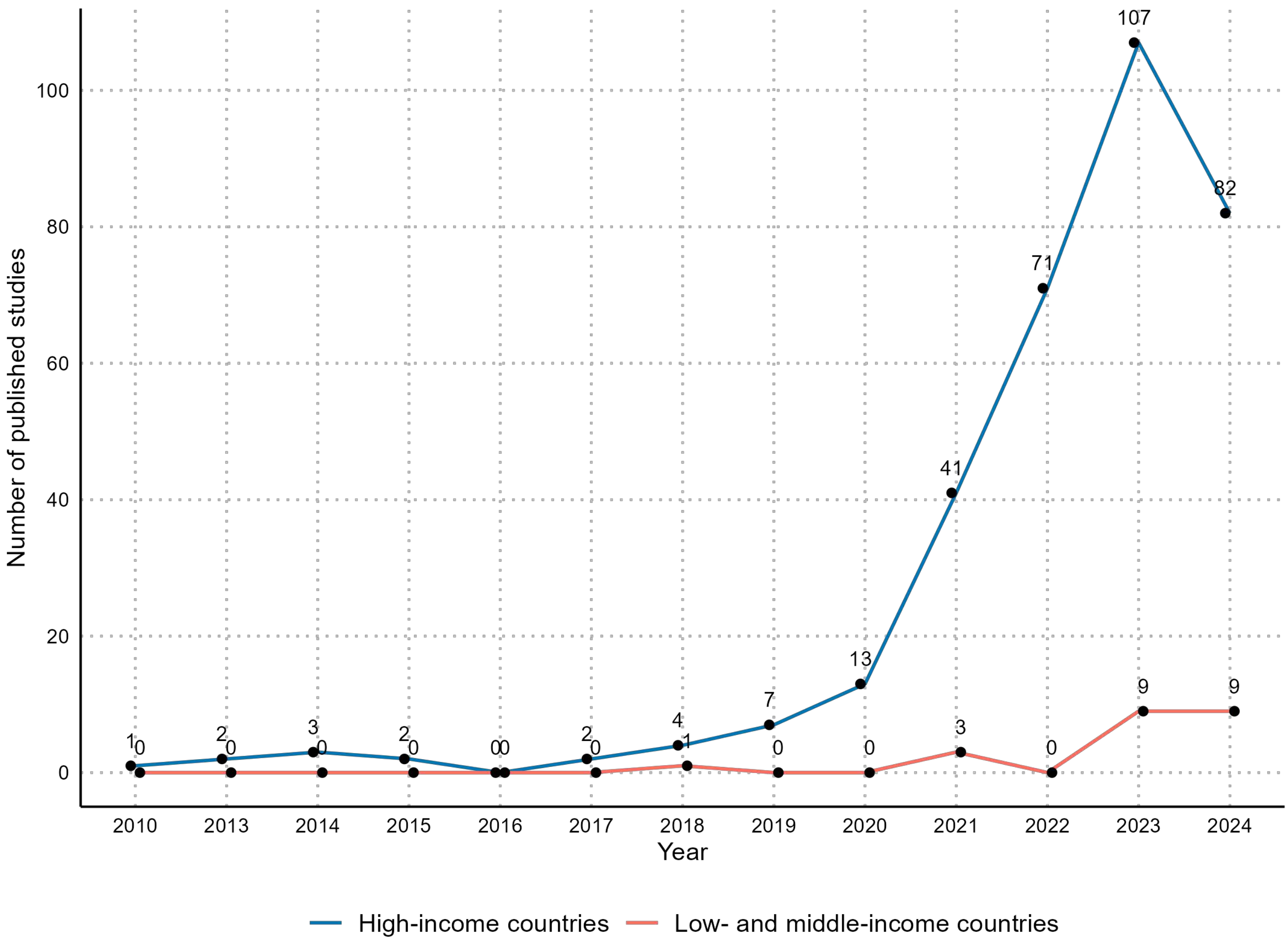

Note: Multi-country studies are not included.

S1 Table. Seach Strategy

N°	SEARCH	HITS
**Cochrane Library (Central Register of Controlled Trials)**		
#1	(((((target* near/2 trial*) AND emulat*))) OR ((("target trial" OR "target* clinical trial") near/3 (emulat* OR mimic* OR simulat* OR pragmatic OR hypothetical))) OR (((pragmatic near/3 emulat*))) OR (((trial* near/3 emulat*))) OR ((((rct* OR "randomised controlled trial" OR "randomized controlled trial") near/3 (emulat* OR hypothetical OR mimic OR mimicking OR mimicked))))):ti,ab,kw	235
#2	MeSH descriptor: [Randomized Controlled Trial] explode all trees	37
#3	MeSH descriptor: [Randomized Controlled Trials as Topic] explode all trees	54940
#4	#2 or #3	54976
#5	(((emulat* or hypothetical or mimic or mimicking or mimicked))):ti	414
#6	#4 and #5	23
#7	#1 or #6	243
**PROSPERO**		
#1	"emulat* trial*" or "trial emulat*"	9
#2	"target trial*" or "target clinical trial*"	61
#3	((pragmatic* or trial* or rct or randomized or randomised) and (emulat* or mimic or mimicking or mimicked or simulat* or hypothetical)):TI,KW	28
#4	#1 OR #2 OR #3	90
**SCOPUS**		
#1	(TITLE-ABS-KEY (((target* W/2 trial*) AND emulat*))) OR (TITLE-ABS-KEY (("target* trial*" OR "target* clinical trial*") W/3 (emulat* OR mimic* OR simulat* OR pragmatic OR hypothetical))) OR (TITLE-ABS-KEY ((pragmatic W/3 emulat*))) OR (TITLE-ABS-KEY ((trial* W/3 emulat*))) OR (TITLE-ABS-KEY (((rct* OR "randomised control* trial*" OR "randomized control* trial*") W/3 (emulat* OR hypothetical OR mimic OR mimicking OR mimicked))))	753
**OVID MEDLINE**		
#1	((target* adj2 trial*) and emulat*).ti,ab,kw.	390
#2	((target* trial* or target* clinical trial*) adj3 (emulat* or mimic* or simulat* or pragmatic or hypothetical)).mp.	374
#3	(pragmatic adj3 emulat*).mp.	8
#4	(trial* adj3 emulat*).mp.	528
#5	((RCT* or randomi?ed control?ed trial*) adj3 (emulat* or hypothetical or mimic or mimicking or mimicked)).mp.	120
#6	randomized controlled trial/ or equivalence trial/ or pragmatic clinical trial/ or exp Randomized Controlled Trials as Topic/	783308
#7	(emulat* or hypothetical or mimic or mimicking or mimicked).ti.	39241
#8	#6 and #7	182
#9	#1 or #2 or #3 or #4 or #5 or #8	754
**EMBASE**		
#1	(target* NEAR/2 trial*) AND emulat*:ti,ab,kw	579
#2	(‘target* trial*’ OR ‘target* clinical trial*’) NEAR/3 (emulat* OR mimic* OR simulat* OR pragmatic OR hypothetical)	529
#3	Pragmatic NEAR/3 emulat*	18
#4	Trial* NEAR/3 emulat*	745
#5	(rct* OR ‘randomi$ed control$ed trial*’) NEAR/3 (emulat* OR hypothetical OR mimic OR mimicking OR mimicked)	102
#6	‘randomized controlled trial’/de OR ‘equivalence trial’/de OR ‘non-inferiority trial’/de OR ‘pragmatic trial’/de OR ‘superiority trial’/de OR ‘randomized controlled trial (topic)’/de	1097560

#7	emulat*:ti OR hypothetical:ti OR mimic:ti OR mimicking:ti OR mimicked:ti	46449
#8	#6 AND #7	343
#9	#1 OR #2 OR #3 OR #4 OR #5 OR #8	1064
